# Multiple health risk behaviors, including high consumption of ultra-processed foods and their implications for mental health during the COVID-19 pandemic

**DOI:** 10.3389/fnut.2022.1042425

**Published:** 2022-11-16

**Authors:** Hillary Nascimento Coletro, Raquel de Deus Mendonça, Adriana Lúcia Meireles, George Luiz Lins Machado-Coelho, Mariana Carvalho de Menezes

**Affiliations:** ^1^School of Nutrition, Research and Study Group on Nutrition and Public Health (GPENSC), Universidade Federal de Ouro Preto, Ouro Preto, Brazil; ^2^Department of Clinical and Social Nutrition, School of Nutrition, Research and Study Group on Nutrition and Public Health (GPENSC), Universidade Federal de Ouro Preto, Ouro Preto, Brazil; ^3^Laboratory of Epidemiology, School of Medicine, Universidade Federal de Ouro Preto, Ouro Preto, Brazil

**Keywords:** anxiety, depression, risk behaviors, fruit and vegetable consumption, ultra-processed food, sedentary behavior

## Abstract

**Background and aim:**

The growing increase in diet- and behavior-related illnesses has drawn the attention of many epidemiologists who attribute such changes to the epidemiological and nutritional transition. Thus, this study aims to evaluate the association between the combined occurrence of health risk behaviors, such as sedentary lifestyles, high weekly consumption of ultra-processed foods (UPFs), and non-daily consumption of fruits and vegetables, and symptoms of anxiety or depression in adults.

**Methods:**

This is a cross-sectional study based on an epidemiological survey in two Brazilian cities. The outcome, anxiety, and depression symptoms were assessed using the Generalized Anxiety Disorder 7-item (GAD-7) and the Patient Health Questionnaire-9 (PHQ-9). Food consumption was assessed using a qualitative food frequency questionnaire (FFQ) with reference to consumption in the last 3 months and categorized into the consumption of fruits and vegetables and the consumption of UPFs according to the NOVA classification. Sedentary behavior was assessed by considering the amount of sitting or reclining time per day reported by participants and categorized as less than 9 h of sitting or reclining and 9 h or more. For the analysis, adjusted Poisson regression (PR) was used to estimate the prevalence ratio and the 95% confidence interval (CI).

**Results:**

Those with the health risk behaviors, non-daily consumption of fruits and vegetables, and high consumption of UPFs had a 2.6 higher prevalence ratio for symptoms of mental disorder (PR: 2.6 and 95%CI: 1.1–6.5), as well as those with all three health risk behaviors, had a 2.8 higher prevalence ratio for symptoms of mental disorder (PR: 2.8 and 95%CI: 1.3–6.1).

**Conclusion:**

This study revealed that the existence of a combination of two and three health risk behaviors led to a higher prevalence of symptoms of anxiety or depression.

## Introduction

According to the International Classification of Diseases (ICD-11), mental disorders are diseases with unusual psychological manifestations that generate functional impairment, and may be the result of biological, social, genetic, physical, or chemical alterations (1). Individuals with mental disorders have a decreased life expectancy of 10–15 years compared to the general population (2). Between 1990 and 2019, the global number of disability-adjusted life-years (DALYs) due to mental disorders increased from 80.8 to 125.3 million, with depression and anxiety being the most prevalent disorders (3).

Depression is defined as a mental disorder in which a sad or irritable mood is present as a common symptom accompanied by cognitive and neurological changes such as difficulty concentrating, sleep disorders, anorexia, and memory changes, (4) while anxiety can be defined as an emotional reaction to aversive situations and can cause somatic manifestations and symptoms such as headache, tachycardia, and tremors, and psychic manifestations such as insecurity, insomnia, and irritability (5). In both cases, studies have shown that hyperactivity of the hypothalamic-pituitary-adrenal axis leads to excessive production of pro-inflammatory cytokines and a decrease in serotonin (6), which are related to lifestyle and health behaviors.

Mental disorders include not only intrinsic determinants such as the ability to cope with thoughts and emotions, but also social, lifestyle, economic, and environmental factors (7). In this scenario, the COVID-19 pandemic was declared in 2020, and negative psychological and behavioral experiences that could elicit extreme psychological stress and contribute to mental health problems were exacerbated. Examples include fear of infection, insecurity about the future, high mortality from SARS-CoV-2 (8), and several health measures enacted to contain high transmission of the disease (9). These measures altered the lifestyle of the entire population, especially work arrangements, social relationships, food consumption, and the practice of physical activity and exercise (10). Thus, the risk factors for the occurrence of disease and mental disorders were exacerbated and the cases of new illness increased. It is estimated that 53.2 million new cases of depression and 76.2 million new cases of anxiety worldwide are diagnosed after the beginning of the COVID-19 pandemic (11). In this regard, it is extremely important to understand how the coexistence of health risk behaviors affects mental health. Health risk behaviors typically have a synergistic effect, and the combination of two or more behaviors generally increases the risk of chronic disease when compared to the presence of each behavior individually (12, 13). Thus, it becomes vital to understand the effects of these combined behaviors on mental health as well.

Some important changes observed in the lifestyle of the population after the beginning of the COVID-19 pandemic refer to food consumption (14) and an increase in sedentary behavior (15). This happened because restrictive measures reduced the frequency of purchases of fresh foods such as fruits and vegetables (16) to the institution of work at home, and interrupted leisure-time physical activity outside the home (17). Also, a significant increase in the consumption of foods not prepared at home and ultra-processed foods (UPFs) due to their price (18), convenience (19), palatability (20), storage (21), and easy access in this health crisis (22). High consumption of UPFs, that is, produced by large-scale industrial processes, and excessive addition of salt, sugar, fat, and substances dedicated to industry (21), can cause vitamin, mineral, and protein deficiency, which can lead to high intake of saturated fat, sugar, salt, ingredients with strong flavor, and chemical additives (23) and is associated with many negative health outcomes including symptoms of anxiety and depression (24). Another expected risk behavior is the increased time in reclining and sitting positions, i.e., sedentary behavior (25). This behavior is associated with a variety of adverse physical health outcomes (26) and may be associated with mental disorders too (17), as people with more time in sedentary behavior experience more symptoms of anxiety and depression (27–29).

Thus, this work aims to evaluate the association between the co-occurrence of health risk behaviors (sedentary behavior, high weekly consumption of UPFs, and non-daily consumption of fruits and vegetables) and the occurrence of anxiety or depression symptoms in adults during the COVID-19 pandemic.

## Materials and methods

### Study design and location

This is a cross-sectional study based on a household epidemiological survey conducted in three stages during a critical moment of the COVID-19 pandemic, October and December of 2020, in two Brazilian cities. This study is part of the “Epidemiological surveillance of COVID-19 in the Inconfidentes Region/MG,” as previously described by Meireles et al. (30).

This study took place in the cities of Ouro Preto and Mariana, Brazil, where a total of 108,170 people live in the urban areas of the two cities with a Municipal Human Development Index (MHDI) of 0.741 and 0.742, respectively (31).

### Study population and sampling

Residents in the urban areas of both cities, more than 18 years old, were considered eligible for this study. The sample size was calculated with the population estimate by the 2010 demographic census (31) for the urban areas, 95% confidence level, design effect equal to 1.5, SARS-CoV-2 infection estimate of 3–10%, and precision, plus a 20% re-composition percentage for any losses, using the OpenEpi tool.

A stratified and cluster sampling design was adopted in three stages: census sector (probability proportional to the number of households), household (systematic sampling), and resident (random), to ensure the representativeness of different socioeconomic strata in the sample. Based on this calculation, 1,789 households were selected and agreed to participate in the study. Of these, 27 individuals were excluded because they did not finish the interview or because they did not collect the blood sample, an inclusion criterion of the survey “Epidemiological surveillance of COVID-19 in the Inconfidentes Region/MG.” Another 46 individuals were excluded with incomplete answers on the scale assessing anxiety symptoms, and another 25 individuals were excluded with incomplete answers on the scale assessing depression symptoms. Therefore, 1,693 individuals were evaluated in this study.

### Data collection

Data collection was conducted on weekends to increase the participation of residents. The process started by approaching households, randomly selecting an adult resident, and drawing lots for the face-to-face interviews.

Interviews were conducted by a trained team, and their health was tracked through periodic evaluation, including testing for anti-SARS-CoV-2. A face-to-face interview lasted around 40 min, using electronic devices.

The questionnaire contained registration data, sociodemographic and economic variables, lifestyle variables, and food consumption assessments.

### Outcome variable: Symptoms of anxiety and depression

The presence of symptoms of anxiety and depression was assessed using two validated scales: the Generalized Anxiety Disorder 7-item (GAD-7) (32) assessed the symptoms of anxiety and Patient Health Questionnaire-9 (PHQ-9) (33) assessed the symptoms of depression.

Both scales included questions that assessed the frequency of situations that triggered the symptoms of anxiety and depression in the last 2 weeks. Each response [(i) none; (ii) several days; (iii) more than half of the days; and (iv) almost every day] has a punctuation that ranges from 0 to 3 points. The points for each answer were added, and the result of this sum was categorized as described by Kroenke et al. (33) and Lowe et al. (32). Thus, scores below 10 points on both scales were considered as minimal or mild symptoms of anxiety and depression, while a score ≥ 10 points was considered as moderate or severe symptoms for both diseases. From this categorization, the binary outcome variable was created, which considers the presence of symptoms of anxiety or depression when the individual achieves a score ≥10 points (32, 33).

### Exposure variables: Food consumption and sedentary behavior

Food consumption was assessed using a qualitative food frequency questionnaire (FFQ), based on the national survey “Surveillance System for Risk and Protective Factors for Chronic Diseases by Telephone Survey (34),” referring to consumption in the last 3 months. The frequency of food consumption was reported on weekdays: [(i) never; (ii) 1–2 days/week; (iii) 3–4 days/week; (iv) 5–6 days/week; and (v) every day, including Saturday and Sunday].

For this study, foods were separated into fruit and vegetable and UPF groups. The consumption of fruits, vegetables, legumes, and dark green vegetables were grouped and categorized into daily consumption and non-daily consumption, and the latter was considered the investigated risk behavior.

**Figure 1:**
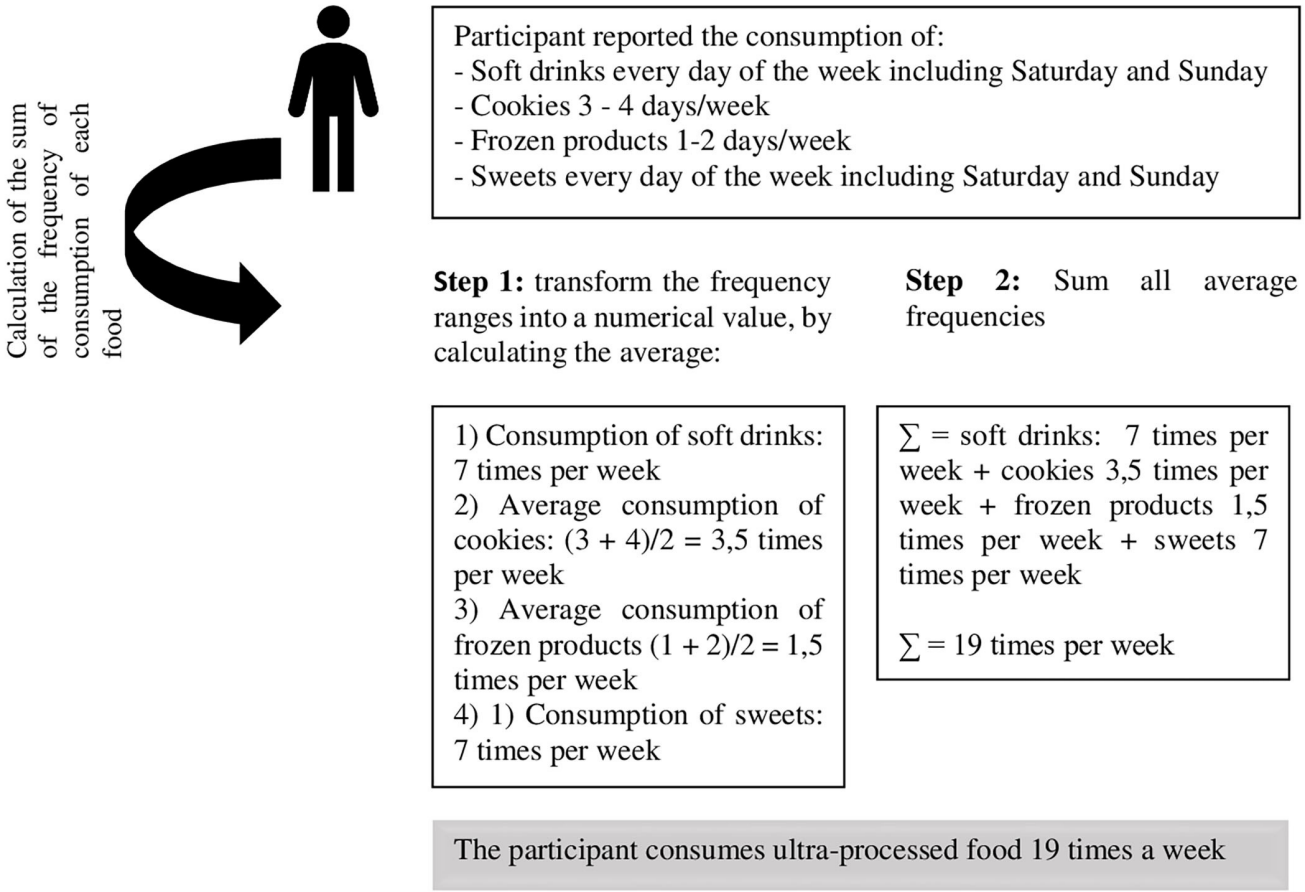
An example of how the exposure variable, high consumption of ultra-processed foods, was created, COVID Inconfidentes 2020.

For the second health risk behavior “high weekly consumption of UPFs,” the foods that make up this group were chosen from the NOVA classification, an internationally recognized instrument that classifies foods based on the extent and purpose of processing and their implications on human health (35). NOVA classifies foods into four groups: (i) fresh or minimally processed foods; (ii) culinary ingredients; (iii) processed foods; and (iv) UPFs (21). For this study, we only used the last group as a proxy for unhealthy eating, UPFs that stands for foods with the highest extent and purpose of processing were formulated with several techniques and many ingredients, including non-natural substances (21). To create this exposure variable, we consider the sum of the weekly consumption frequencies of all UPFs in the FFQ: soft drinks, chocolate drinks and artificial yogurt, cookies, packaged snacks, instant noodles, frozen products, processed meats, sweetbreads, and sweets (Figure 1), considering that these are the most consumed UPFs in the country (36, 37). Then, we categorized this variable into consumption below the average weekly frequency (<15.15 times/week) and consumption equal to or above the average weekly frequency (≥15.15 times/week), and the last one was considered the risk behavior under investigation.

Sedentary behavior was assessed by considering the amount of time sitting or reclining per day (38) reported by participants. Based on data from a recent meta-regression analysis of more than 1 million participants that suggest a cut-off point for sedentary behavior, this variable was categorized into less than 9 h of sitting or reclining time and 9 h or more of sitting or reclining time per day, and the last one was considered the risk behavior under investigation (39, 40).

### Covariates

Sociodemographic variables were investigated to describe the sample and explore possible confounding. Sociodemographic variables investigated were sex, age (grouping: 18–34, 35–59, and 60 years old or more), marital status (with or without a partner), skin color (white, black/brown, indigenous, yellow and others, in which the participant could mention another color not previously mentioned), education (never attended school, 1–9 years of study, or more than 9 years of study), family income (up to two minimum wages MW, two to four MW, or more than four MW), employed or not at the time of the interview, and change in income after the COVID-19 pandemic (reduced, increased, or no change).

### Statistical analysis

Initially, we calculated the sample weight of each selected unit (census sector, household, and individual) separately for each city to increase the representativeness of the sample.

For descriptive analysis, the proportion and 95% confidence intervals (CIs) were used. To evaluate the relationship between the descriptive variables and the outcome, Pearson's Chi-squared test was used with a 5% significance level.

To confirm the hypothesis of an association between health risk behaviors and symptoms of anxiety or depression, a variable was created, which was made up of the sum of the health risk behaviors ranging from 0 to 3, where 0 indicated no health risk behaviors, 1 indicated the presence of one of the assessed health risk behaviors (non-daily fruits and vegetables, having high consumption of UPF, and having a sedentary behavior), 2 indicated the presence of two of the assessed health risk behaviors, and 3 indicated the presence of all of the assessed health risk behaviors. A multivariate analysis was performed using Poisson regression (PR) with the prevalence ratio and respective 95%CI for binary outcomes.

In addition, eight patterns of health risk behaviors were created: (i) having none of the studied health risk behaviors (a pattern that can be used as a reference for analysis); (ii) having only non-daily fruit and vegetable consumption as a health risk behavior; (iii) having only high UPF consumption as a health risk behavior; (iv) having only sedentary behavior as a health risk behavior; (v) non-daily fruits and vegetables and having high UPF consumption; (vi) non-daily fruits and vegetables and having sedentary behavior; (vii) having high consumption of UPF and sedentary behavior; and (viii) non-daily fruits and vegetables, having high consumption of UPF, and having sedentary behavior, and additive interaction analysis was used to verify whether there are differences in prevalence ratios from the combination of different health risk behaviors for symptoms of anxiety or depression. An additive interaction is defined as a differential reduction in the absolute risk associated with one factor between different levels of other factors, and aims to assess the attributable risk estimate based on absolute differences between prevalence ratios (41, 42).

**Figure 2:**
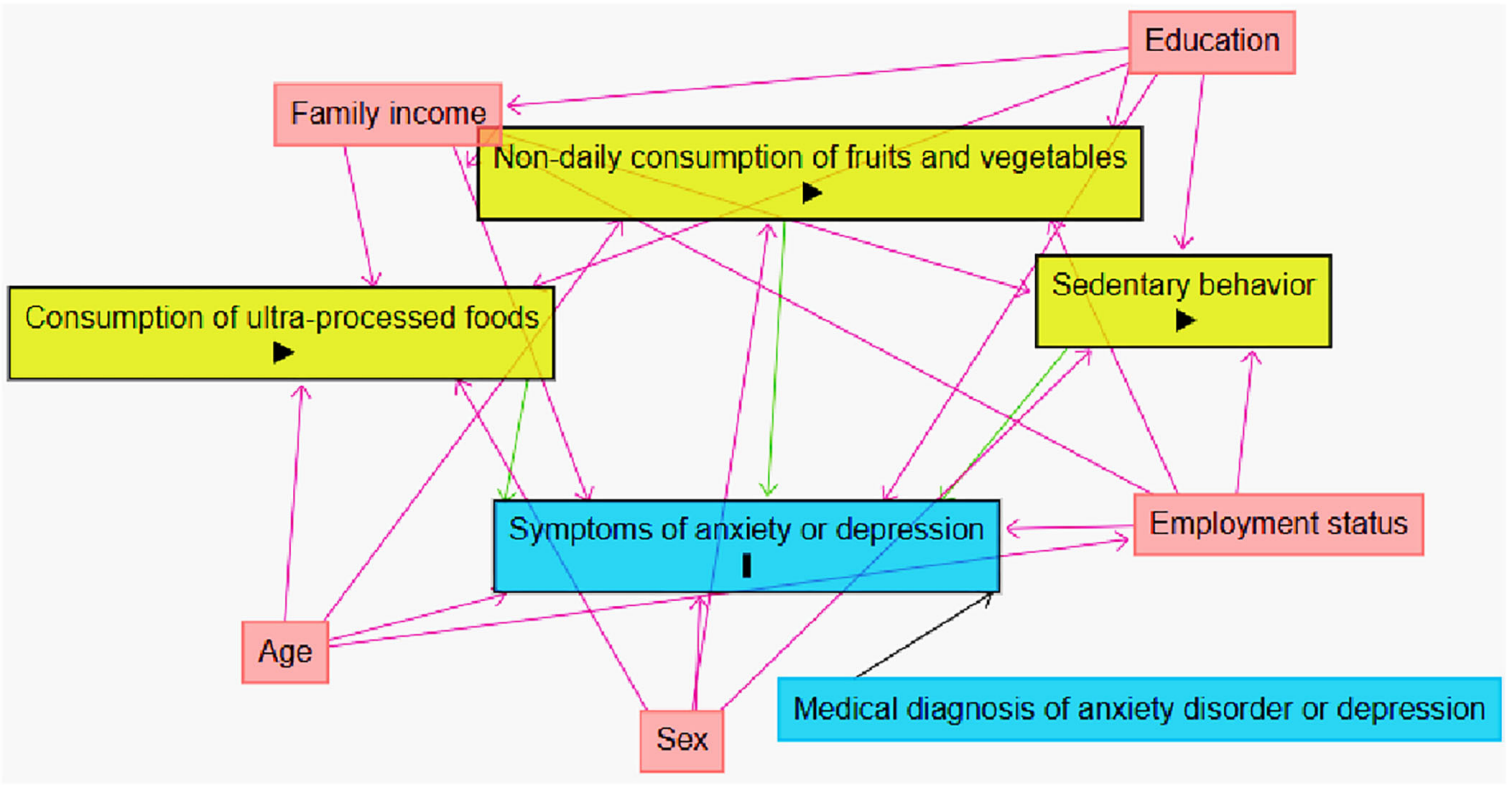
Directed Acyclic Graph (DAG) for the association between anxiety or depression symptoms and health risk behavior, with possible confounding variables, COVID Inconfidentes 2020. Causal connections are represented by arrows; 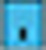 represents the outcome; 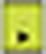 represents the exposure variables; 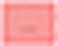 represents the adjustment variables.

To select appropriate adjustment variables, we created a Directed Acyclic Graph (DAG) (Figure 2), considering exposures (non-daily fruit and vegetable consumption, high weekly consumption of UPF, and sedentary behavior), the outcome (anxiety or depression symptoms), and possible confounding variables. To avoid unnecessary adjustments, a minimal and sufficient set of adjustment variables was defined: sex, age, family income, employment status, and education.

The analysis was performed using Stata software version 15.1 (Stata Corporation, College Station, Texas), using the command “svy,” which considers a complex sample design.

### Ethics approval

The study was approved by the Research Ethics Committee of the Universidade Federal de Minas Gerais (Protocol No. 4.135.077), and all participants signed the written informed consent.

## Results

**Figure 3:**
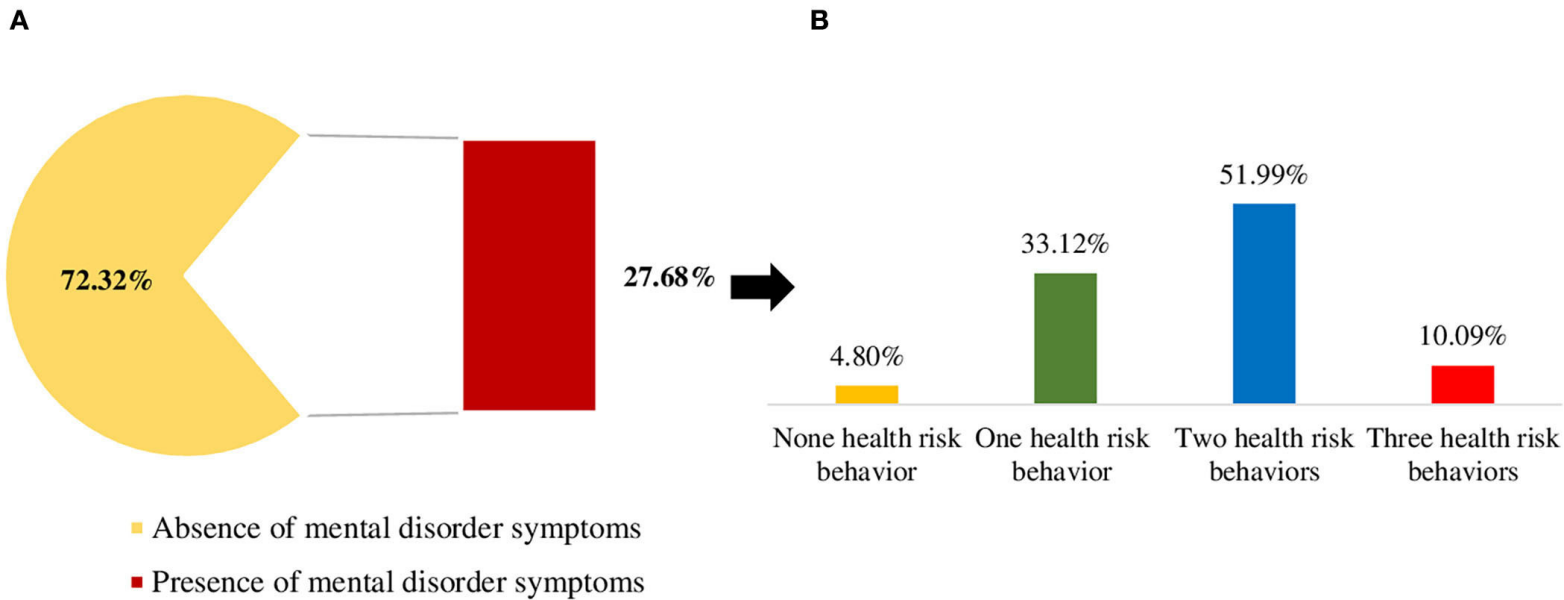
Prevalence of health risk behaviors among individuals with symptoms of anxiety or depression, COVID Inconfidentes 2020. **(A)** Prevalence of mental disorder symptoms. **(B)** Prevalence of the combined occurrence of health risk behaviors among those with mental disorder symptoms. The health risk behaviors: sedentary behavior (≥9 h/day), non-daily consumption of fruits and vegetables, and high weekly consumption of ultra-processed foods (≥15.15 times per week).

Of the participants, 1,693 were eligible for this study, and 27.6% reported symptoms of anxiety or depression (Figure 3).

The majority of the samples were women (51.1%), aged 35 to 59 years (45.8%), married (53.4%), brown or black in skin color (69.5%), had studied more than 9 years (77.4%), and had a family income less than twice the minimum wage (40.5%). In addition, most of them reported that they had a job during the interview period (52.9%) and that their income had not changed since the beginning of the COVID-19 pandemic (54.9%) (Table 1). Among those with symptoms of anxiety or depression, 18.5% reported sedentary behavior, 91.4% did not consume fruits and vegetables daily, and 55.3% consumed larger amounts of UPFs, as indicated by the abovementioned average weekly frequency.

**Table 1:** Sociodemographic and mental health characteristics according to the outcome, the presence of symptoms of anxiety and depression, COVID Inconfidentes 2020.

**Variables**	**Total % (95%CI)**		
		**Absence of symptoms of mental disorder**	**Presence of symptoms of mental disorder**	P- value^*^
**Sex** ^**a**^				0.001
Male	48.90 (41.67–56.18)	54.00 (46.45–61.36)	35.59 (24.62–48.30)	
Female	51.10 (43.82–58.33)	46.00 (38.64–53.55)	64.41 (51.70–75.38)	
**Age** ^**a**^				0.144
18 to 34 years	36.52 (31.92–41.38)	33.88 (27.39–41.05)	43.41 (31.94–55.64)	
35 to 59 years	45.87 (41.34–50.47)	46.08 (39.39–52.91)	45.33 (35.47–55.58)	
≥ 60 years	17.61 (14.46–21.28)	20.04 (16.00–24.80)	11.26 (7.79–15.99)	
**Marital status** ^**a**^				0.071
Married	53.41 (47.38–59.34)	55.86 (50.27–61.30)	47.01 (36.27–58.03)	
Not married	46.59 (40.66–52.62)	44.14 (38.70–49.73)	52.99 (41.97–63.73)	
**Skin color** ^**a**^				0.275
White	26.10 (20.98–31.97)	23.93 (18.38–30.54)	31.73 (22.28–42.99)	
Brown and black	69.53 (63.23 - 75.17)	71.63 (65.44–77.11)	64.05 (52.57–74.12)	
Indigenous, yellow and others	4.37 (2.88–6.59)	4.43 (2.48–7.82)	4.21 (2.27–7.69)	
**Education** ^**a**^				0.369
Never attended school	1.53 (0.06–3.54)	1,16 (0.03–0.37)	0.25 (0.07 −0.79)	
1 to 9 years	21.05 (17.15–25.57)	22.29 (17.69–27.68)	17.81 (12.56–24.63)	
> 9 years	77.41 (72.69–81.53)	76.54 (71.03–81.29)	79.69 (72.17–85.58)	
**Family income** ^**a**^				0.497
≤ 2 MW^**b**^	40.59 (35.20–46.21)	42.08 (34.70–49.84)	36.70 (26.98–47.64)	
> 2 to ≤ 4 MW^**b**^	31.99 (26.81–37.67)	32.35 (26.98–38.24)	32.26 (20.39–46.98)	
> 4 MW^**b**^	27.42 (22.41–33.08)	25.56 (19.81–32.32)	31.03 (23.26–40.06)	
**Working status** ^**a**^				0.549
Be employed	52.95 (48.09–57.75)	54.18 (47.09–61.10)	49.74 (39.50–60.00)	
Be unemployed	47.05 (42.25–51.91)	45.82 (38.90–52.91)	50.26 (40.00–60.50)	
**Change in income after the COVID-19 pandemic** ^**a**^				0.100
Yes, it has reduced	37.29 (31.81–43.12)	36.71 (31.11–42.70)	38.80 (29.51–48.98)	
Yes, it has increased	7.79 (4.59–12.91)	5.31 (3.17–8.75)	14.26 (5.22–33.43)	
No change	54.92 (50.57–59.20)	57.98 (51.33–64.35)	46.94 (36.28–57.88)	
**Health risk factors**				
**Sedentary behavior** ^**a**^				0.292
Yes	15.44 (12.30–19.21)	14.22 (11.03–18.15)	18.56 (11.76–28.05)	
No	81.44 (71.95–88.24)	85.78 (81.85–88.97)	81.44 (71.95–88.24)	
**Non-daily fruit and vegetable consumption** ^**a**^				0.008
Yes	83.87 (78.56–88.07)	80.95 (72.79–87.10)	91.49 (87.86–94.11)	
No	16.13 (11.93–21.44)	19.05 (12.90–27.21)	8.51 (5.89–12.14)	
**High weekly consumption of ultra-processed foods** ^**a**^				0.009
Yes	42.62 (38.49–46.86)	37.73 (32.42–43.36)	55.31 (44.55–65.60)	
No	57.38 (53.14–61.51)	62.27 (56.64–67.58)	44.69 (34.40–55.45)	

a Values expressed as proportion and 95% confidence interval (CI);

b MW: Minimum wage of the year when data collection occurred, 2020—BRL 1,045.00 or about USD 194;

* Statistically significant *p*-values; Not married, Widowed, divorced, and single.

When evaluating the combined occurrence of health risk behaviors, we observe that among individuals affected by symptoms of depression or anxiety, 4.8% had not received an assessment of health risk behavior, 33.1% had one health risk behavior, 51.9% had two health risk behaviors, and 10.0% had all three health risk behaviors at the same time (Figure 3).

**Figure 4:**
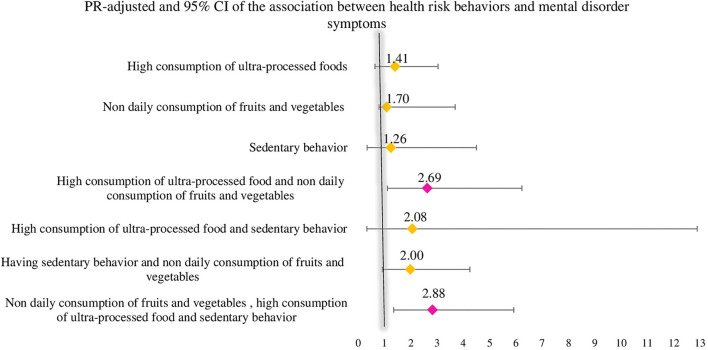
Prevalence ratio and 95% confidence interval (CI) for the interaction analysis between the co-occurrence of health risk behaviors and the presence of symptoms of mental disorder, COVID Inconfidentes 2020. Combined analysis to verify the existence of an association between the outcome (presence of depression or anxiety symptom) and the explanatory variables, given as health risk behaviors sedentary behavior, non-daily consumption of fruits and vegetables, and high weekly consumption of ultra-processed foods. The analysis was adjusted for sex, age, family income, having a job, and education.

In a multivariate regression analysis that combined the three health risk behaviors (sedentary behavior, non-daily consumption of fruits and vegetables, and high consumption of UPFs) with the presence of symptoms of anxiety or depression (Figure 4), it was possible to identify that those who combined diet-related risk behaviors (non-daily consumption of fruits and vegetables and high consumption of UPFs) had a 2.6 higher PR for symptoms of mental disorder (PR: 2.6 and 95% CI: 1.1–6.5), and those who engaged in the three risk behaviors simultaneously had a PR 2.8 higher for symptoms of mental disorder (PR: 2.8 and 95% CI: 1.3–6.1).

**Figure 5:**
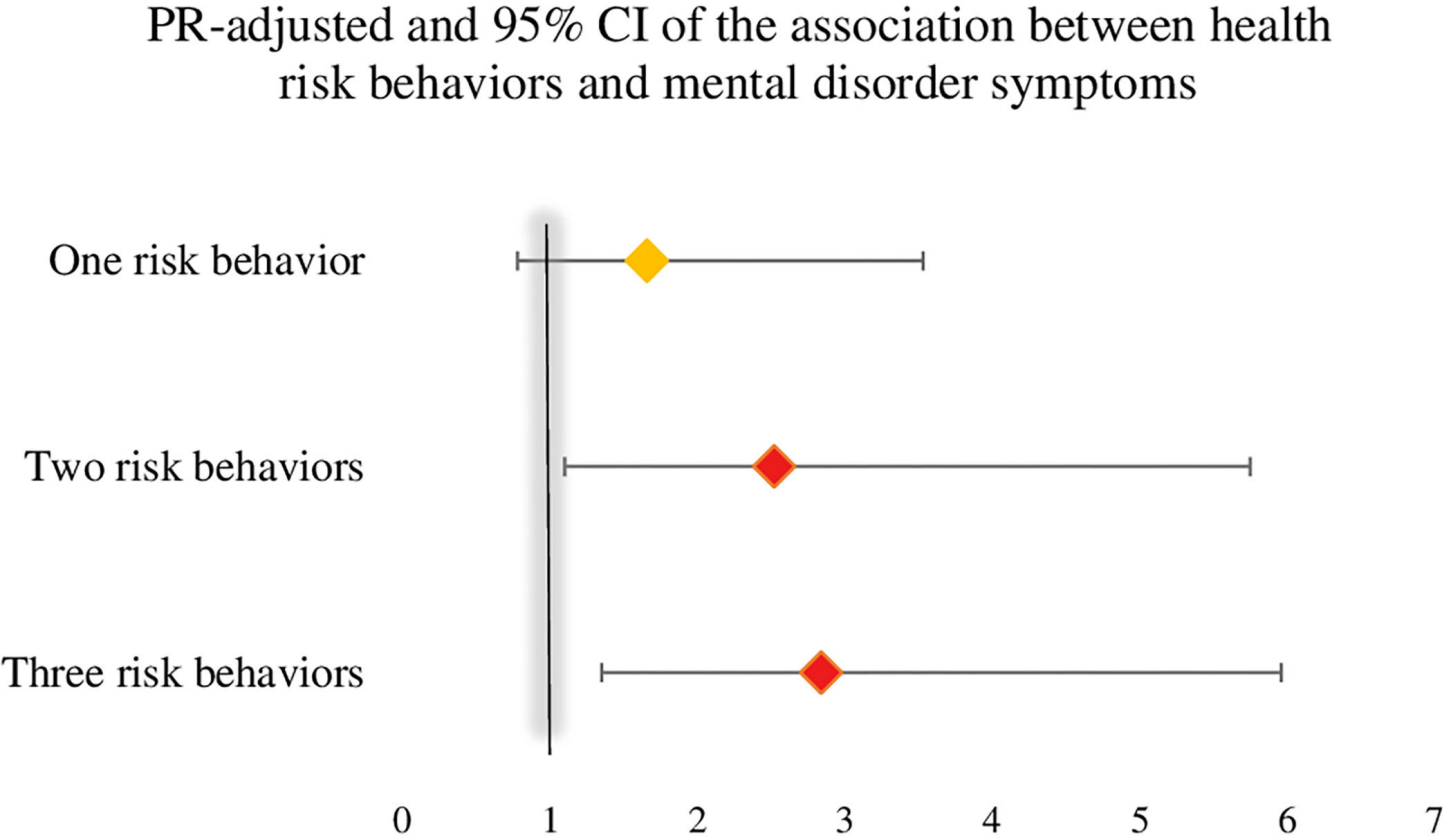
Prevalence ratio and 95% CI for the association between the co-occurrence health risk behaviors and the presence of symptoms of mental disorder, COVID Inconfidentes 2020. Poisson regression analysis to verify the existence of an association between the outcome (presence of depression or anxiety symptom) and the explanatory variables, given as health risk behaviors sedentary behavior, non-daily consumption of fruits and vegetables, and high weekly consumption of ultra-processed foods. The analysis was adjusted for sex, age, family income, having a job, and education.

Furthermore, in a multivariate regression analysis of the association between the co-occurrence of health risk behaviors and the presence of symptoms of anxiety or depression (Figure 5), those with two and three health risk behaviors were observed to have a 2.5 and 2.8 higher PR for symptoms of mentaldisorder during the COVID-19 pandemic (PR: 2.5 and 95% CI: 1.1–6.0/ PR: 2.8 and 95%CI: 1.3–6.1).

## Discussion

This study revealed a high prevalence of mental disorder symptoms, given the high prevalence of symptoms of anxiety and depression in Brazilian adults during the COVID-19 pandemic. This prevalence may be explained by the fear of the disease and its implications for health, uncertainties about the spread of the virus and its treatment, the high mortality rates, and the loss of family members and relatives, in addition to the disruption of daily routines and lifestyles marked by the imposition of restrictive measures characterized by social distance (8), the restriction of physical and in-person trade (43), the decrease in physical activity during leisure time and the increase in sedentary behavior (44, 45), increased alcohol consumption (46, 47), a decreased purchase of fresh foods, and increased impulse buying (48), especially of ready-to-eat foods with high durability (49, 50), characterizing health risk behaviors. Studies have shown that engaging in these behaviors, especially those related to comfort, may be a way for people to manage psychological distress and stressful situations (51, 52).

The isolated prevalence of health risk behaviors, especially those associated with mortality and the development of chronic non-communicable diseases, has been a widely explored theme; however, there are few studies that seek to investigate the combination of health risk behaviors and their association with mental health in a pandemic context, to identify the different lifestyle patterns and the possible synergistic effects of these behaviors (12, 13). Usually, health risk behaviors have a synergistic effect, changing the influence of one behavior on another. Furthermore, it is understood that lifestyle-related behaviors share contextual determinants, acting directly on the development of negative habits that lead to illness (52). Thus, the data presented here, have important implications for public health, as they help to identify which health risk behaviors grouped together, aiding in the development of an integrative approach for effective interventions and targeted initiatives for the prevention of mental health conditions.

Our data reveal that the combined risk behaviors, non-daily consumption of fruits and vegetables, and high consumption of UPFs had a higher prevalence ratio for symptoms of mental disorder. Epidemiological evidences suggest a relationship between food consumption and poor mental health through inflammatory reactions and deficiency of nutrients and neurotransmitters (53, 54). Excessive consumption of carbohydrates and sugar has been described as a risk factor for mental disorders due to an increase in neuroinflammation (55). Moreover, in addition to the macronutrient composition of UPFs, their non-natural ingredients, such as additives, colorings, flavorings, and sweeteners, induce changes in the human microbiota that can cause intestinal dysbiosis and mediate inflammatory processes that start in the gut and extend to the brain and can disrupt the production of important neurotransmitters responsible for feelings of wellbeing and happiness (56, 57).

In contrast, the consumption of fibers, present in larger quantities in fruits and vegetables, is associated with good overall health, as they are metabolized into short-chain fatty acids, which are important anti-inflammatory agents (58). In addition, these fresh foods are good sources of complex vitamins B, D, and E, and play an import role in modulating brain functions related to cognitive performance, preventing neurodegenerative disorders, and protection against oxidative stress (59). A survey of our group that evaluates food consumption according to the degree of processing and symptoms of anxiety and depression showed an inverse association between a higher consumption of fresh/minimally processed foods and the prevalence of depression symptoms, as well as a direct association between a higher consumption of UPFs and a higher prevalence ratio of depression symptoms (24, 60, 61).

When evaluating diet-related health risk behaviors combined with sedentary behavior, a higher prevalence ratio was observed for mental disorder symptoms. Sedentary behavior has been studied as a risk factor for mental illness as screen-based sedentary behaviors, such as the use of computer, television (TV), and social media, are likely to induce addiction and poor sleep quality, which can maximize levels of mental distress (15, 62). Furthermore, it is suggested that the greater the time spent in sedentary behavior, the less social interaction and, therefore, the greater the feeling of loneliness and sadness (63, 64). Another biological mechanism explaining the association between sedentary behavior and mental disorders is that increased screen exposure in sitting or reclining time can reduce serum brain-derived neurotrophic factor, which in normal amounts is associated with cardiovascular health, cognitive development, and good mental health (65). A meta-analysis showed that after the beginning of the COVID-19 pandemic, children increased their time spent sitting or reclining by approximately 159.5 min/day, while adults increased their time spent in sedentary behavior by 126.9 min/day, which was negatively correlated with overall mental health, depression, anxiety, and quality of life (66).

In this regard, this paper adds data that demonstrate scientific evidence on the health consequences, in addition to SARS-CoV-2 infection, derived from the COVID-19 pandemic, highlighting the urgent need for public policies capable of jointly controlling health risk behaviors, such as the regulation of the production and sale of UPFs, to promote policies to improve food quality and health. The ingredients in ultra-processed products make them sugary or salty, often high in saturated fats or trans fats, and poor in micronutrients and other bioactive compounds, which are associated with many negative health outcomes (23). Thus, fiscal policies (67–69) warning labels (70, 71), marketing restrictions (72, 73), and incentives to consume fresh/minimally processed foods (21) are fundamental and should be the next steps to control UPF consumption, as guided by the World Health Organization (WHO), which recommends the consumption of five servings of fruits and vegetables per day (74).

Sedentary behaviors can be influenced by environmental attributes in specific contexts. Evidence suggests that it is important to increase the number of breaks in sedentary time, stand up and move after 30 min of uninterrupted sitting, for example, when watching TV or using a computer, and to replace leisure time sitting or reclining by time spent in physical activity (38, 75). As well as it is necessary to regulate public policies that can mitigate sedentary behaviors, either by reformulating urban settings encouraging more physical exercise practices, such as bike paths, walking trails, and parks, and reformulating the policies that refer to workers' health because most sedentary behaviors occur during the workday (76, 77).

Despite the significant findings, this study has some limitations. First, it is a cross-sectional study, which does not allow causal inferences to be established. The explanatory variables of food consumption were measured from a qualitative point of view, with information on the weekly frequency of their consumption, without the possibility of numerically quantifying the consumption. However, the use of a qualitative FFQ with the most consumed foods by the study population is a very important method to report the quality of the diet in general. Sedentary behavior was categorized based on a cut-off point proposed by a meta-analysis as it does not have an official measure. In addition, the outcome was assessed according to the presence of symptoms of anxiety or depression, measured by scales, and not by medical diagnosis, and the possibility of misclassification, if the participant did not answer correctly. However, the scales used have been validated (78, 79). Meanwhile, it is important to highlight that a robust methodology was used during a difficult time of the pandemic, considering that face-to-face interviews allow greater accuracy of the information obtained, while the probabilistic sample selection and sample weight provided statistical power to the study.

In conclusion, this study revealed that the existence of a combination of two and three health risk behaviors led to a higher prevalence of symptoms of anxiety or depression, considering that diet-related risk behaviors as a whole stood out as an important risk for mental disorder symptoms. We suggest the use of multi-behavioral interventions as a promising strategy for managing multifactorial morbidities (such as mental disorders), especially when considering the complexity of behaviors associated with individual lifestyle (80, 81). In addition, it should be considered as the institution and regulation of public policies aimed at structuring an urban setting to allow the population to exercise and live a healthy lifestyle, with full access to establishments for the production and sale of natural foods and places for physical activity and exercise. Furthermore, we reinforce the importance of the construction of guidelines based on the Food Guide for the Brazilian Population (21) and the Physical Activity Guide for the Brazilian population (82), to control high consumption of UPFs, encourage the consumption of natural foods, reduce sedentary behavior, and encourage physical activity, especially in the post-pandemic period.

## Data availability statement

The raw data supporting the conclusions of this article will be made available by the authors upon request, without undue reservation.

## Ethics statement

The studies involving human participants were reviewed and approved by Research Ethics Committee of the Universidade Federal de Minas Gerais (Protocol No. 4.135.077). The patients/participants provided their written informed consent to participate in this study.

## Author contributions

HC: data collection supervision, conception and study design, analysis and interpretation of data, writing the manuscript, critical review, and final approval. RM: analysis and interpretation of data, critical review, and final approval. AM and GM-C: conception and coordination of data collection, critical review, management of financial resources, and final approval. MM: conception and study design, analysis and interpretation of data, critical review, supervision, and final approval. All authors contributed to the article and approved the submitted version.

## Funding

This work was supported by the Universidade Federal de Ouro Preto, Fundação de Amparo à Pesquisa do Estado de Minas Gerais, Edital No. 001/2021—APQ-02445–21, Conselho Nacional de Desenvolvimento Científico e Tecnológico and Coordenação de Aperfeiçoamento de Pessoal de Nível Superior (CAPES), funding code: CAPES 09/2020 - No. 88881.504995/2020-0188881.504995/2020-01.

## Conflict of interest

The authors declare that the research was conducted in the absence of any commercial or financial relationships that could be construed as a potential conflict of interest.

## Publisher's note

All claims expressed in this article are solely those of the authors and do not necessarily represent those of their affiliated organizations, or those of the publisher, the editors and the reviewers. Any product that may be evaluated in this article, or claim that may be made by its manufacturer, is not guaranteed or endorsed by the publisher.
